# The**** Clinical Characteristics and Prediction Nomograms for Primary Spine Malignancies

**DOI:** 10.3389/fonc.2021.608323

**Published:** 2021-02-26

**Authors:** Lei Zhou, Runzhi Huang, Ziheng Wei, Tong Meng, Huabin Yin

**Affiliations:** ^1^ Department of Orthopedics, Shanghai General Hospital, Shanghai Jiao Tong University School of Medicine, Shanghai, China; ^2^ Shanghai Bone Tumor Institution, Shanghai, China; ^3^ Division of Spine, Department of Orthopedics, Tongji Hospital Affiliated to Tongji University School of Medicine, Shanghai, China

**Keywords:** primary spine malignancy, clinical characteristic, prognostic factor, nomogram, survival

## Abstract

**Background:**

Primary spine malignancies (PSMs) are relatively rare in bone tumors. Due to their rarity, the clinical characteristics and prognostic factors are still ambiguous. In this study, we aim to identify the clinical features and proposed prediction nomograms for patients with PSMs.

**Methods:**

Patients diagnosed with PSMs including chordoma, osteosarcoma, chondrosarcoma, Ewing sarcoma, and malignant giant cell tumor of bone (GCTB) between 1975 and 2016 were selected from the Surveillance, Epidemiology, and End Results (SEER) database. The patient and tumor characteristics were described based on clinical information. The significant prognostic factors of overall survival (OS) and cancer-specific survival (CSS) were identified by the univariate and multivariate Cox analysis. Then, the nomograms for OS and CSS were established based on the selected predictors and their accuracy was explored by the Cox–Snell residual plot, area under the curve (AUC) of receiver operator characteristic (ROC) and calibration curve.

**Results:**

The clinical information of 1,096 patients with PSMs was selected from the SEER database between 1975 and 2016. A total of 395 patients were identified with full survival and treatment data between 2004 and 2016. Chordoma is the commonest tumor with 400 cases, along 172 cases with osteosarcoma, 240 cases with chondrosarcoma, 262 cases with Ewing sarcoma and 22 cases with malignant GCTB. The univariate and multivariate analyses revealed that older age (Age > 60), distant metastasis, chemotherapy, and Surgery were independent predictors for OS and/or CSS. Based on these results, the nomograms were established with a better applicability (AUC for CSS: 0.784; AUC for OS: 0.780).

**Conclusions:**

This study provides the statistics evidence for the clinical characteristics and predictors for patients with PSMs based on a large size population. Additionally, precise prediction nomograms were also established with a well-applicability.

## Introduction

Primary spine malignancies (PSMs) are relatively rare in bone tumors with a wide histopathological heterogeneity (1, 2). Chordoma is the most common tumor type, with the predominate sites of fusion segments (3). Besides, chondrosarcoma, Ewing sarcoma, osteosarcoma, and malignant giant cell tumor of the bone (GCTB) can be commonly found in the spine (4, 5). Due to the specific location of the spine, these tumors often result in local pain, neurologic defect, spinal instability and pathological fracture, which significantly decrease the life quality and overall survival (OS) (6). Till now, surgery is still the main therapeutic method for spine malignancies. As for the surgical treatment, the total *en-bloc* spondylectomy (TES) is recommended. However, due to the specific structure of the spine, many cases are unsuitable for TES, thereby leading to poor prognosis. Thus, there is a pressing need for oncologists and orthopedists to identify the epidemiology features and prognostic factors for patients with PSMs.

Many previous studies have focused on the clinical characteristics and OS-associated factors of PSMs, such as age, Karnofsky performance score (KPS), pathological nature, Frankel grading and therapeutic methods (7–9). However, due to their rarity, most of these studies were based on small sample size in one single tumor center, and their epidemiological features are not well known. In addition, although many score systems of spine metastasis have been constructed, few of them can be applied in PSMs (10–12). Thus, their clinical features and predictors should be identified by a large cohort of patients and advanced modeling methods should also be used to establish a well-applied prediction model.

The Surveillance, Epidemiology, and End Results (SEER) database is a widely used database consisting clinical data of cancer patients (13). Previous studies focused on the clinical features and predictors of PSMs in SEER database were only based on one tumor type with a relative short period, which limit their reliability (13–15). The nomogram is a visual statistical predictive tool for identifying clinically relevant prognostic factors. Thus, recent study has utilized this tool to predict the prognosis specific to an individual and achieves satisfying results (13, 16, 17).

In this study, we selected patients with PSMs from the SEER database with a long period (from 1975 to 2016) to explore their clinical characteristics. In addition, patients from 2004 to 2016 with full treatment information were used to identify the prognostic factors. Based on the identified prognostic factors by regression analysis methods, the nomograms were constructed to evaluate the OS and cause specific survival (CSS) of each individual (15). In general, our finding may identify the comprehensive clinical features of patients with PSMs and provide the well applicable nomograms.

## Methods

### Patient Selection and Data Extraction

Patients diagnosed with spine malignancy (Osteosarcoma, Chondrosarcoma, Chordoma, Ewing sarcoma, and GCTB) between 1975 and 2016 were selected from the SEER database. Only patients whose tumor diagnosed with the first primary tumor by histopathological evidence were included in the epidemiological analysis. The clinical data included demographics (*i.e.* age, gender and ethnicity), tumor characteristics (*i.e.* histologic subtype, SEER historic stage, and tumor extension), treatment information (*i.e.* surgery, radiation, and chemotherapy). The end events were defined as all-cause death and cause specific death. Thus, the evaluation indicator consisted of OS time, OS status, CSS time, and CSS status. The patients with unknown demographics, tumor and treatment information as well as outcomes were excluded from further survival analysis. Especially, all ICD-O-3 histologic subtypes were combined for five tumor types and different subtypes with similar survival outcomes were also combined for survival analysis.

### Statistical Analysis

The epidemiological analysis was described as demographics, tumor characteristics, treatment information and patient outcomes. All of them were presented by integrated bar-plot and heatmap in each ICD-O-3 histologic subtype.

To identify potential variables for prognosis, Kaplan–Meier survival analysis and Cox regression analysis were used and were presented by Kaplan–Meier curve and forest plot, respectively. The significant variables of the univariate analysis were screened out to construct the multivariate Cox regression model for OS and CSS. Despite the non-significant demographic recodes, demographics were all kept for subsequent multivariate analysis because clinical variables require correction of this information. Furthermore, the prognostic nomograms were constructed based on the multivariate Cox models to predict the 3- and 5-year OS and CSS probability of patients with PSMs.

In the multivariate Cox regression model, the formula was used to calculate the risk score for each individual:

$$risk\;score_{N}\;=\;\beta 1\;\times {\mathrm{variable}}_{1}+\beta 2\;\times {\mathrm{variable}}_{2}+\ldots \ldots +\beta M\times {\mathrm{variable}}_{M}$$

In the formula, “N” represented the number of each patient; “*β*” represented coefficient of each variable in the multivariate model; and “M” represented the number of prognostic variables in the multivariate model.

All patients were subsequently divided into high and low risk groups with the median of the risk score. Kaplan–Meier survival analysis and risk scatter/line plot were utilized to evaluate the independent prognosis value of the risk score and risk distribution in patients with PSMs, respectively. In terms of model diagnosis, the good of fitness (GOF), discrimination and calibration of the multivariate Cox regression model was illustrated by the Cox–Snell residual plot, area under the curve (AUC) of receiver operator characteristic (ROC) and calibration curve, respectively.

### Statistical Method

Statistical integration started with descriptive statistic: dichotomous variables were summarized as percentages, and continuous variables were reported as mean (range). Two-sided P value <0.05 was applied to identify the statistically significant variables in this study. In the Cox regression model, the significance was described by hazard ratio (HR) and 95% confidence interval (CI). The R software (version 3.6.2, www.r-project.org, Institute for Statistics and Mathematics, Vienna, Austria) was used for all statistics analysis processes.

## Results

### Patient Selection and Data Extraction

**Figure 1:**
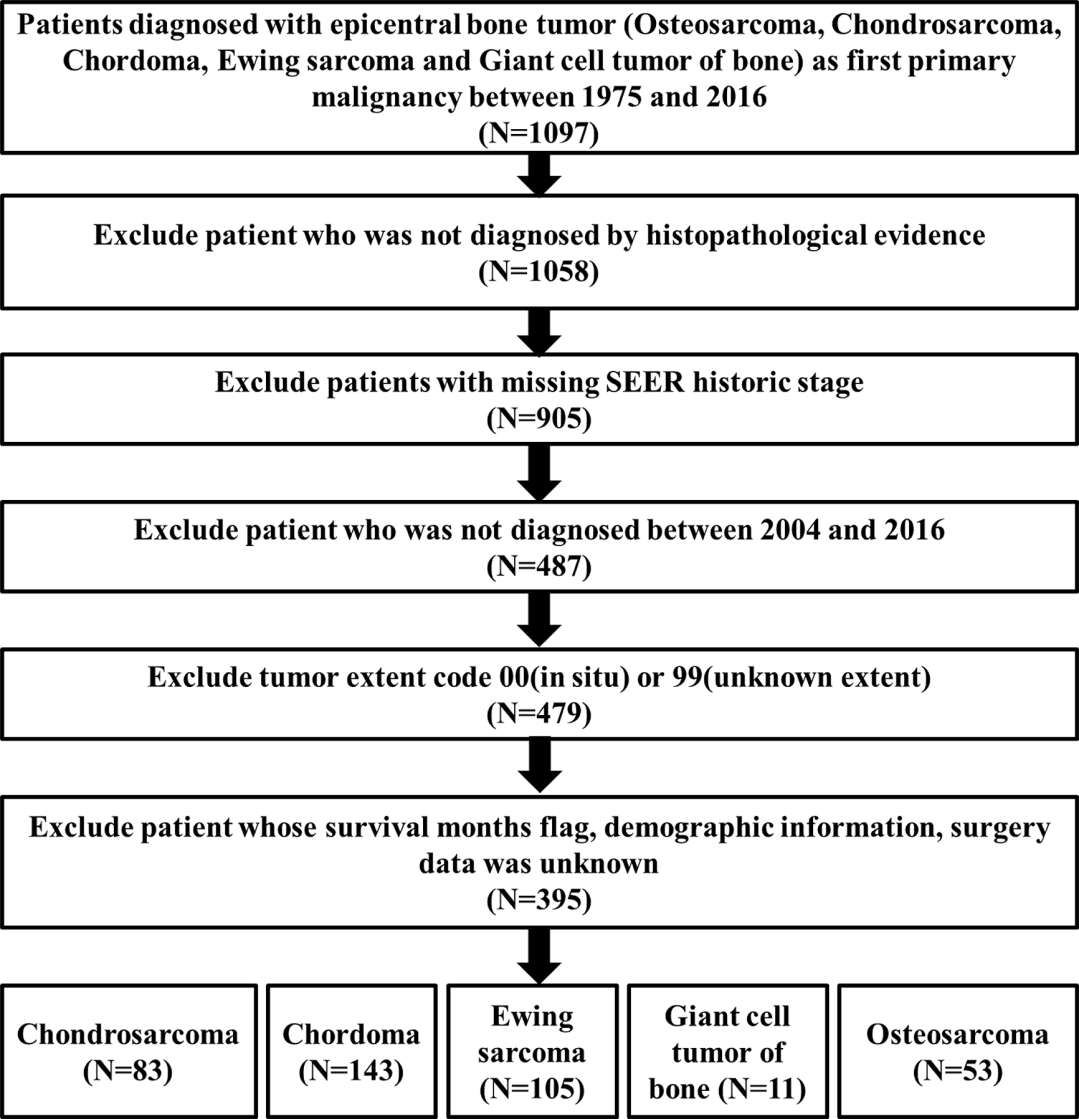
The flowchart of inclusion and exclusion process.

The flowchart of inclusion and exclusion processes was summarized in **Figure 1**. A total of 1,097 patients diagnosed with PSMs between 1975 and 2016 were selected from the SEER database. After excluding patients who were not diagnosed by histopathological evidence, 1,058 patients were subsequently included in the epidemiological analysis.

### Descriptive Epidemiological Statistic

**Figure 2:**
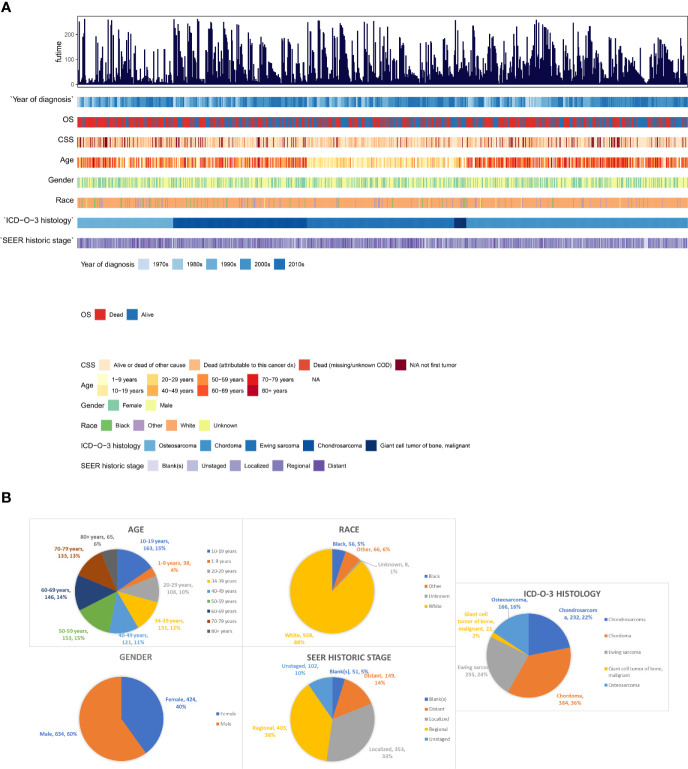
The epidemiological analysis of 1,058 patients with PSMs. **(A)** The integrated bar-plot and heatmap of demographics, tumor information and patient outcomes of patients with PSMs. **(B)** The pie chart of age, race, gender, historic stage and ICD-O-3 histology in the patients with PSMs. PSMs, primary spine malignancies.

All demographics, tumor information and outcomes of all the 1,058 patients were summarized by integrated bar-plot and heatmap (**Figure 2A**). The total cohort comprised 634 (60%) males and 928 (88%) white patients, with a similar age distribution from 10- and 79-year old (**Figure 2B**). Regional tumors (403, 38%) and localized tumors (353, 33%) are common in those PSMs, and 149 (14%) patients experienced tumor metastasis (**Figure 2B**). As for tumor histology (**Figure 2B**), chordoma was the most common pathological pattern (384, 36%). Ewing sarcoma (355, 24%) and chondrosarcoma (232, 22%) were similarly distributed. Osteosarcoma was found in 166 patients (16%), whereas malignant giant cell tumor of bone (21, 2%) was rarely uncovered.

**Figure 3:**
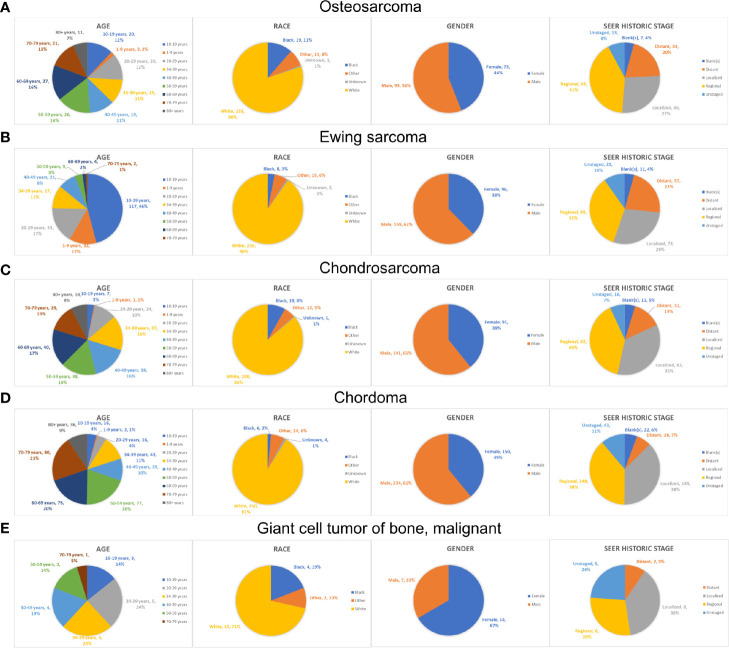
The epidemiological analysis of patients with different types of PSMs. The pie chart of age, race, gender and historic stage in the patients with osteosarcoma **(A)**, Ewing sarcoma **(B)**, chondrosarcoma **(C)**, chordoma **(D)** and malignant GCTB **(E)**. PSMs, primary spine malignancies; GCTB, Giant cell tumor of bone.

As the tumor histology is an important clinical data which is significantly associated with patients’ prognosis, we further summarized the demographics data of each ICD-O-3 histologic subtype (Osteosarcoma, **Figure S1A**; Ewing sarcoma, **Figure S1B**; Chondrosarcoma, **Figure S1C**; Chordoma, **Figure S1D**; malignant GCTB, **Figure S1E**). As for age, patients with osteosarcoma/chondrosarcoma/malignant GCTB had a similar distribution of age, and few patients were younger than 10-years old (**Figures 3A, C, E**). Patients with Ewing sarcoma were relatively young, with 12% younger than 10-years old and 46% in the second decade (**Figure 3B**). However, most chordoma patients (70%) were older than 50-years old and even 9% patients were older than 80-years old (**Figure 3D**). Due to the data from the SEER database, most patients of each tumor type were white race (**Figures 3A-E**). In addition, patients with osteosarcoma/Ewing sarcoma/chondrosarcoma/chordoma were male predominance, whereas most malignant GCTB (67%) were female (**Figures 3A–E**). Furthermore, we uncovered that chordoma and malignant GCTB rarely metastasized, while osteosarcoma and Ewing sarcoma had a high tendency of distant metastasis (**Figures 3A, B, D, E**).

### Survival Analysis of Selected PSM Patients

To further obtain full treatment data of surgery and adjuvant therapy (radiotherapy and chemotherapy), we selected the chordoma patients treated from 2004 to 2016. Finally, a total of 395 patients with PSMs were identified to decipher the prognostic factors, including 53 osteosarcomas, 83 chondrosarcomas, 143 chordomas, 105 Ewing sarcomas, and 11 malignant giant cell tumors of bone (**Figure 1**). In these 395 patients, 327 underwent surgery, along with 230 having radiotherapy and 143 experiencing chemotherapy. Due to the correlation between tumor type/surgery and adjuvant therapy, we also identified their relation. The results revealed that there was significant difference between patients undergoing radiotherapy with and without surgery (P = 0.516, **Figure S2A** and **Table S1**). More patients treated with chemotherapy and surgery than those undergoing chemotherapy without surgery (P < 0.001, **Figure S2B** and **Table S2**). Besides, more patients with chordoma or Ewing sarcoma were treated with radiotherapy (P < 0.001, **Figure S2C** and **Table S3**) and more patients with Ewing sarcoma or osteosarcoma underwent chemotherapy (P < 0.001, **Figure S2D** and **Table S4**).

**Figure 4:**
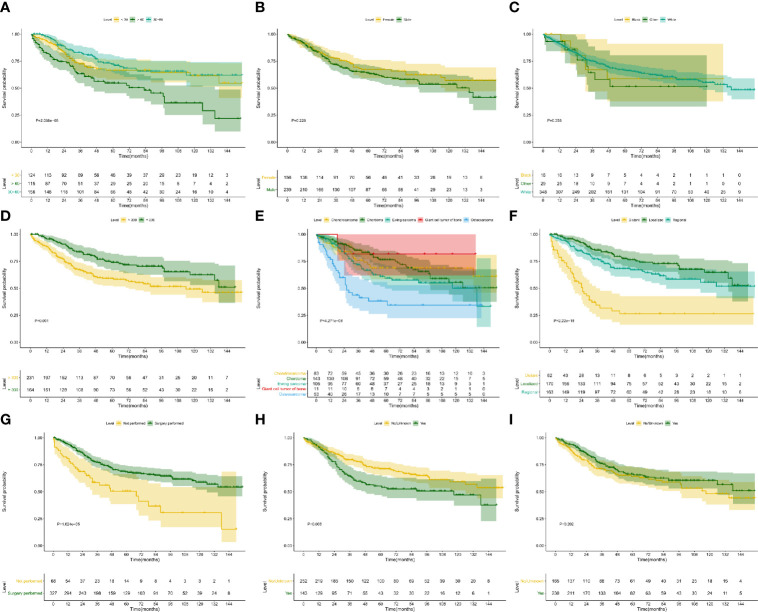
Kaplan–Meier survival analysis for CSS. The Kaplan–Meier survival curves of age (**A**, P < 0.001), gender (**B**, P = 0.228), race (**C**, P = 0.358), extension (**D**, P = 0.001), ICD-O-3 histology (**E**, P < 0.001), SEER historic stage (**F**, P < 0.001), surgery (**G**, P < 0.001), chemotherapy (**H**, P = 0.006) and radiotherapy (**I**, P = 0.202) for CSS. CSS, cancer-specific survival.

**Figure 5:**
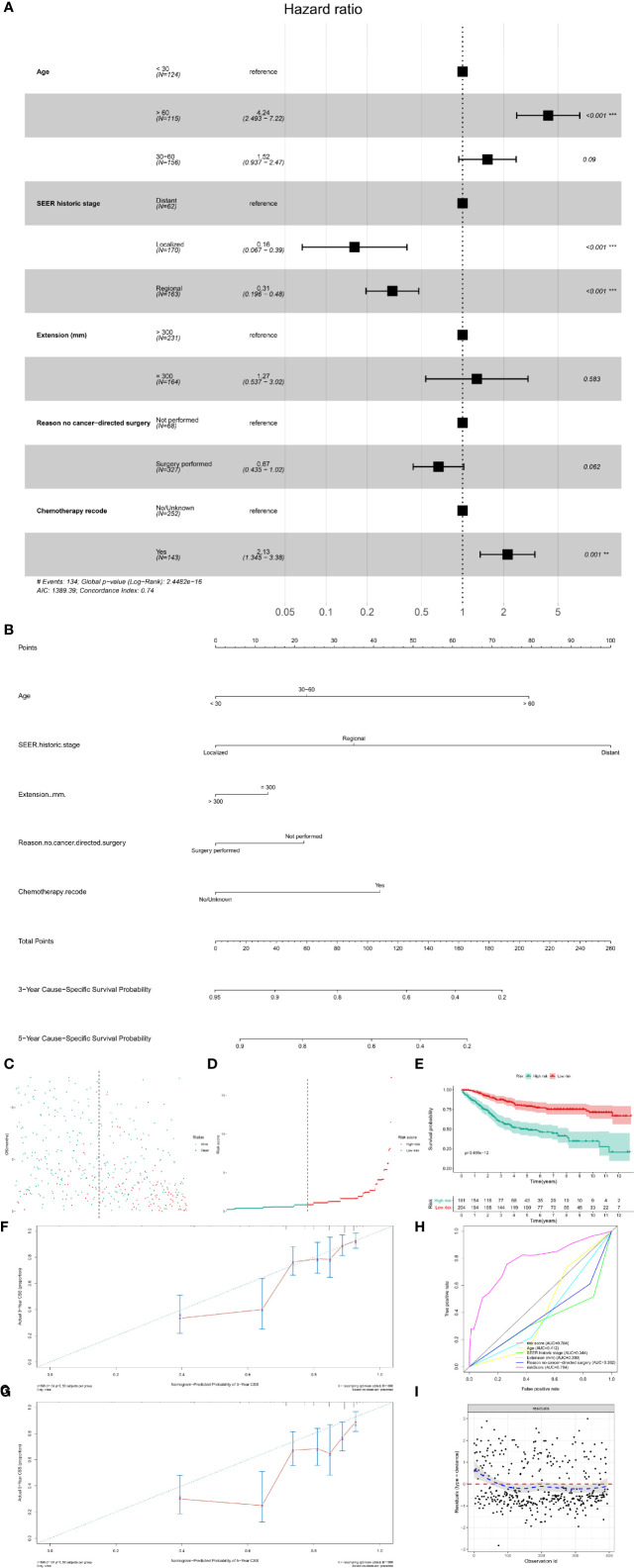
The multivariate Cox regression model and nomogram for CSS. **(A)** The multivariate Cox regression analysis for CSS; **(B)** The prognostic nomogram for CSS. The risk line **(C)** and scatterplot **(D)** of the risk score. The Kaplan–Meier survival curve of the risk score for CSS (**E**, P < 0.001). The calibration curve **(F, G)**, ROC curve **(H)** and Cox–Snell residual plot **(I)** of the multivariate Cox regression model for CSS. CSS, cancer-specific survival; ROC, receiver operator characteristic.

The Kaplan–Meier survival analysis was used to evaluate the prognostic values of age (**Figure 4A**, P < 0.001), gender (**Figure 4B**, P = 0.228), race (**Figure 4C**, P = 0.358), extension (**Figure 4D**, P = 0.001), ICD-O-3 histology (Figure 4E, P < 0.001), SEER historic stage (**Figure 4F**, P <0.001), surgery (**Figure 4G**, P < 0.001), chemotherapy (**Figure 4H**, P = 0.006) and radiotherapy (**Figure 4I**, P = 0.202) for CSS. Six prognostic factors were identified and analyzed in the multivariate Cox regression. The results revealed that patients with older age (Age > 60: HR, 4.24; 95% CI, 2.49–7.22; P < 0.001; Reference, Age < 30) and chemotherapy (HR, 2.13; 95% CI, 1.345–3.38; P = 0.001; Reference, No/Unknown) were significant risk variables for CSS. Besides, patients with localized tumor (HR, 0.16; 95% CI, 0.067–0.39; P < 0.001; Reference, Distant) and regional tumor (HR, 0.31; 95% CI, 0.196–0.48; P < 0.001; Reference, Distant) were significant favorable variables for CSS (**Figure 5A**).

Based on the multivariate Cox models, the prognostic nomogram was constructed, which could predict the 3- and 5-year CSS probability of patients with PSMs (**Figure 5B**). The risk score for each patient was calculated by the formula described in the *Methods* section. The risk line and scatterplot illustrated the distribution of risk score among all the patients, respectively (**Figures 5C, D** **)**. In addition, the Kaplan–Meier survival curve showed a significantly prognostic value of the risk score for CSS (**Figure 5E**, P < 0.001). In terms of model diagnosis, the calibration curve, ROC curve (AUC = 0.784) and Cox–Snell residual plot showed acceptable calibration, discrimination and GOF and of the multivariate Cox regression model for CSS (**Figures 5F–I**).

**Figure 6:**
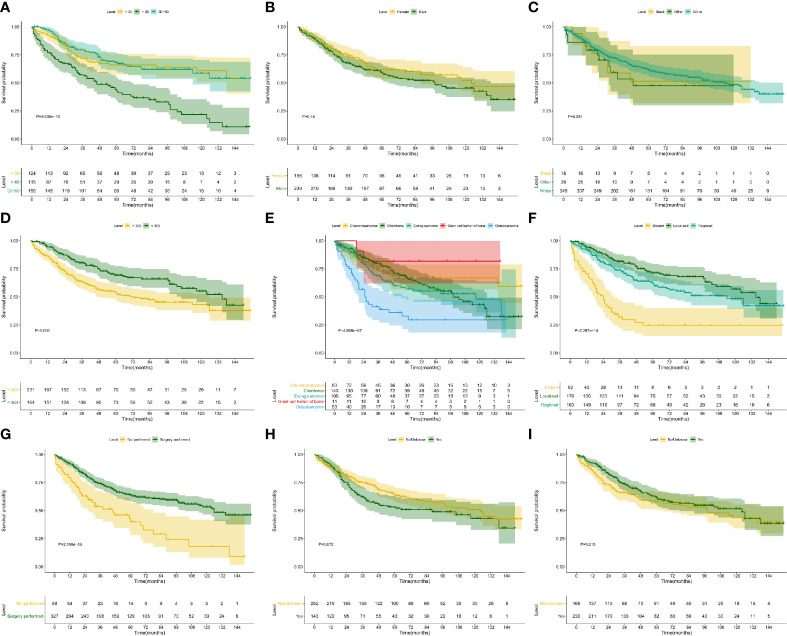
Kaplan–Meier survival analysis for OS. The Kaplan–Meier survival curves of age (**A**, P < 0.001), gender (**B**, P = 0.180), race (**C**, P = 0.261), extension (**D**, P = 0.001), ICD-O-3 histology (**E**, P < 0.001), SEER historic stage (**F**, P < 0.001), surgery (**G**, P < 0.001), chemotherapy (**H**, P = 0.072) and radiotherapy (**I**, P = 0.215) for OS. OS, overall survival.

**Figure 7:**
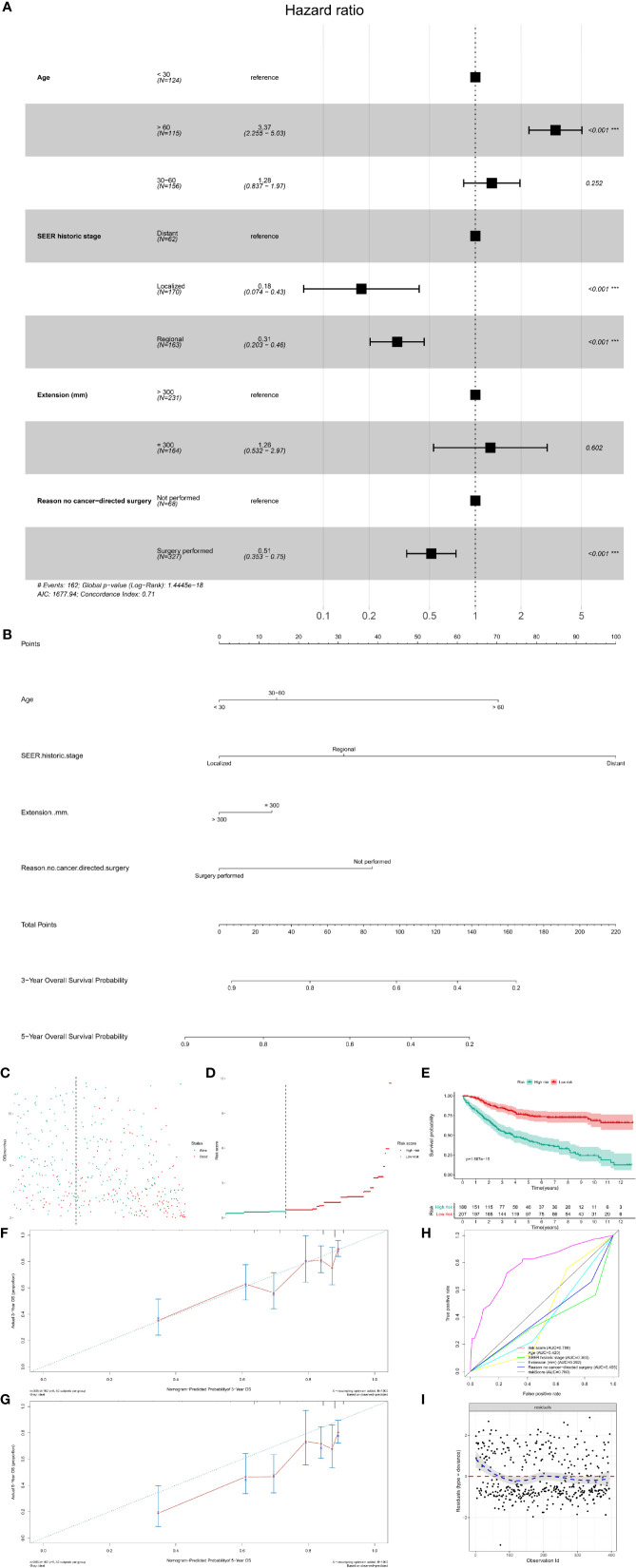
The multivariate Cox regression model and nomogram for OS. **(A)** The multivariate Cox regression analysis for OS; **(B)** The prognostic nomogram for OS. The risk line **(C)** and scatterplot **(D)** of the risk score. The Kaplan–Meier survival curve of the risk score for OS **(E**, P < 0.001**)**. The calibration curve **(F, G)**, ROC curve **(H)** and Cox–Snell residual plot **(I)** of the multivariate Cox regression model for OS. OS, overall survival; ROC, receiver operator characteristic.

Similar to CSS survival analysis, the Kaplan–Meier method was also used to evaluate the predictors for OS. The same six factors were identified as prognostic ones, namely age (P < 0.001), extension (P = 0.001), ICD-O-3 histology (P < 0.001), SEER historic stage (P < 0.001), surgery (P < 0.001), and chemotherapy (P = 0.072). Their Kaplan–Meier curves for OS were illustrated in **Figure 6**. In the multivariate Cox regression analysis for OS, patients with older age (age > 60: HR, 3.37; 95% CI, 2.255–5.03; P < 0.001; Reference, age < 30) was significant risk variables for OS. In addition, compared with patients with distant tumor, those with localized (HR, 0.18; 95% CI, 0.074–0.43; P < 0.001) or regional tumor (HR, 0.31; 95% CI, 0.203–0.46 P < 0.001) have a favorable prognosis. Furthermore, surgical treatment significantly improved the OS of patients with PSMs (HR, 0.51; 95% CI, 0.353–0.75; P < 0.001; **Figure 7A**).

The prognostic nomogram was also constructed to predict the 3- and 5-year OS probability of patients with PSMs (**Figure 7B**). The risk line and scatterplot of OS illustrated the distribution of risk score among all patients (**Figures 7C, D** **)**. In the Kaplan–Meier survival analysis, risk score for OS revealed a significant prognostic value (**Figure 7E**, P < 0.001). To evaluate the calibration, discrimination, and GOF of the multivariate Cox regression model for OS, calibration curve, ROC curve, and residual plot were used. The former revealed a suitable calibration (**Figures 7F, G** **)**; the AUC of ROC was 0.780 (**Figure 7H**); the latter showed a good GOF (**Figure 7I**).

### Subgroup Cox Regression Analysis With Time Frame of Diagnosis

Although some previous epidemiological study comparing SEER areas with non-SEER areas in the United States concluded that the age and sex distributions of these areas were comparable, we could not agree with them about the temporal and spatial heterogeneity of the SEER data any more (18–20). However, to further minimize this temporal and spatial heterogeneity, we also performed four subgroup Cox regression analysis and conducted four nomograms with time frame of diagnosis [(Patients diagnosed between 2004 and 2010 for OS, **Figure S3**); patients diagnosed between 2004 and 2010 for CSS, **Figure S4**); patients diagnosed between 2011 and 2016 for OS, **Figure S5**); patients diagnosed between 2011 and 2016 for CSS, **Figure S6**)]. All models were diagnosed by calibration, time-related ROC and decision curve, suggesting the significant predictors were stable at different times.

## Discussion

PSMs are rare tumors with poor prognosis (21). Due to the specific spinal structure, surgery, and radiotherapy may damage the normal function of the spinal cord and result in neurologic defects (22). Thus, their therapeutic options are challenging. Evaluating the clinical features and prognostic factors may assist orthopedists in early diagnosis and treatment decision-making, whereas those have not been explored comprehensively. Based on the SEER database, we found that chordoma is the commonest tumor type with 36% of all the PSMs, followed by Ewing sarcomas (24%) and chondrosarcoma (22%). Patients with Ewing sarcomas had a younger age, whereas chordoma patients were relatively old. Malignant GCTB had a female predominance, and osteosarcoma and Ewing sarcomas had a high tendency to metastasize. In addition, patients with older age (Age > 60) or distant metastasis had poor prognosis for both CSS and OS. Chemotherapy is an unfavorable factor of the patients’ CSS. Surgery could significantly improve the OS of patients with PSMs.

Generally, the age of PSM patients is not consistent with previous studies, and it is largely associated with the tumor histology (3, 4, 23). In this study, PSM patients had a similar age distribution from 10- and 79-year old, whereas only 10% patients were diagnosed younger than 10-year old or older than 79-year old. In addition, Ewing sarcoma patients were relatively young, and 58% patients were younger than 20-years old. Chordoma patients were relatively old, and most chordoma patients (70%) were older than 50-years old. Furthermore, we also found that old age was an unfavorable predictor for both OS and CSS. This might be due to the poor physical condition of old patients, which could impair the tolerance of operation and subsequently decrease the survival time. With regard to patients’ demographics, gender and race are also common features. We uncovered a slight male predominance (60%) in PSM patients. However, most patients with malignant GCTB were female. As the collected data came from SEER database, white was the leading race a matter of course.

There are many tumor types of PSMs, such as bone tumors, soft tissue sarcoma, and neurogenic tumor (6, 24). In this study, we mainly investigated the bone tumors of PSMs, and we identified five histological types in SEER database. Chordoma consisted of 36% PSMs and were regarded as the most common one. Malignant giant cell tumor of the bone was a rare type of PSM, with only 21 patients being identified. Although histological type was not the prognostic factor in the nomogram for CSS and OS, the prognosis of patients with different histological types were varied. Based on the Kaplan–Meier survival analysis, we found that patients with chordoma, chondrosarcoma or Ewing sarcomas had similar prognosis, which was more favorable than osteosarcoma patients and poorer than malignant GCTB patients.

Surgical treatment is the standard treatment strategy for PSMs, and it provides many benefits, such as tumor resection, local pain relief, spinal cord decompression, and spinal reconstruction (21, 25). In this study, we found that surgical treatment could significantly improve the OS of PSM patients, indicating its important roles in the control of PSM. As for tumor resection, the resection method and margin condition are also critical factors in the final outcome of PSM patients (22, 26). Compared to subtotal excision, *en-bloc* tumor resection with wide margins (R0), recommended by many spine surgeons, offers long-term disease control for most PSMs (27–29). However, the treatment information in the SEER database does not include these data which limits the power of similar studies (15, 30).

Both chemotherapy and radiotherapy are widely used as adjuvant therapies for the clinical management of spine malignancies. As for ES and osteosarcoma, chemotherapy is the standard treatment method and recommended to be performed preoperatively and postoperatively (31). Besides, radiotherapy is often used in ES, and unresectable or recurrent OS, chondrosarcoma and malignant GCTB (31). However, both chemotherapy and radiotherapy only provide limited benefits for chordoma (32). In this study, we found the poor prognosis in PSM patients treated with chemotherapy. In the subgroup analysis, most chemotherapy-applied patients were osteosarcoma and Ewing sarcomas. Thus, we supposed that the poor prognosis of chemotherapy-applied patients may be associated with the tumor histology.

Although this study provides a comprehensive analysis for the clinical features and prognostic factors of PSM patients, there are still some limitations. First, as a population-based study from SEER database, some important data associated with patients’ prognosis are missing, such as resection mode, chemotherapy strategy and radiotherapy dose. Second, it has all the limitations inherent in retrospective studies (Retrospective studies have lower level of evidence than prospective studies in the theory of evidence-based medicine). Third, all the cases in this study are from America, thus data from Europe and Asia are still needed to verify our results. Last but not least, due to the limitation of SEER database, surgical margin status, chemotherapy and radiotherapy strategy were incomplete. To ensure the missing values did not impact outcomes, several subgroup analyses were performed based on the dataset separated the missing values. In the future, more important variables would be collected and incorporated into the nomogram. As our future direction, some vital genetic or epigenetic signatures associated with these risk indicators, which has been validated by multi-omics data and wet experimental assays will be integrated to develop a more rigorous nomogram.

## Conclusion

This study provides the statistics evidence for the clinical characteristics and predictors for patients with PSMs based on a large size population. Additionally, precise prediction nomograms were also established with a well-applicability.

## Data Availability Statement

Publicly available datasets were analyzed in this study. This data can be found here: Surveillance, Epidemiology, and End Results (SEER) database (https://seer.cancer.gov/).

## Author Contributions

LZ: Data collection, Writing - original draft. RH: Data analysis, Writing - original draft. ZW: Data collection, Writing - original draft. HY: Methodology, Software. TM: Conceptualization, Supervision, Writing - review & editing. All authors contributed to the article and approved the submitted version.

## Funding

This study was supported in part by the National Natural Science Foundation of China (No. 81702659; 81772856; 82073207), The Youth Fund of Shanghai Municipal Health Planning Commission (No. 20174Y0117), Interdisciplinary Program of Shanghai Jiao Tong University (No.YG2017MS26), Shanghai Talent Development Fund (No.2018094).

## Conflict of Interest

The authors declare that the research was conducted in the absence of any commercial or financial relationships that could be construed as a potential conflict of interest.

## Supplementary Material

Supplementary Figure 1 The epidemiological analysis of patients with different types of PSMs. The integrated bar-plot and heatmap of demographics, tumor information, and patient outcomes of patients with osteosarcoma **(A)**, Ewing sarcoma **(B)**, chondrosarcoma **(C)**, chordoma **(D)** and malignant GCTB **(E)**. PSMs, primary spine malignancies; GCTB, Giant cell tumor of bone.

Supplementary Figure 2 The subgroup analysis between radiotherapy/chemotherapy and surgery/tumor histology. The bar graph revealed the correlation between radiotherapy and surgery **(A)**, between radiotherapy and tumor histology **(B)**, between chemotherapy and surgery **(C)**, between chemotherapy and tumor histology **(D)**.

Supplementary Figure 3 Construction of the prognostic nomogram with 179 patients diagnosed between 2004 and 2010 for predicting OS. **(A)** The constructed prognostic nomogram based on the multivariate Cox model. **(B)** The calibration curve illustrated decent calibration of the prognostic nomogram. The decision curve **(C)** and time-related ROC **(D)** were also conducted to show the patient benefit and the discrimination of the nomogram.

Supplementary Figure 4 Construction of the prognostic nomogram with 179 patients diagnosed between 2004 and 2010 for predicting CSS. **(A)** The constructed prognostic nomogram based on the multivariate Cox model. **(B)** The calibration curve illustrated decent calibration of the prognostic nomogram. The decision curve **(C)** and time-related ROC **(D)** were also conducted to show the patient benefit and the discrimination of the nomogram.

Supplementary Figure 5 Construction of the prognostic nomogram with 216 patients diagnosed between 2004 and 2010 for predicting OS. **(A)** The constructed prognostic nomogram based on the multivariate Cox model. **(B)** The calibration curve illustrated decent calibration of the prognostic nomogram. The decision curve **(C)** and time-related ROC **(D)** were also conducted to show the patient benefit and the discrimination of the nomogram.

Supplementary Figure 6 Construction of the prognostic nomogram with 216 patients diagnosed between 2004 and 2010 for predicting OS. **(A)** The constructed prognostic nomogram based on the multivariate Cox model. **(B)** The calibration curve illustrated decent calibration of the prognostic nomogram. The decision curve **(C)** and time-related ROC **(D)** were also conducted to show the patient benefit and the discrimination of the nomogram. (Page 14, Lines 14–30; Page 15, Lines 1–6).
