# Supplementary figures and images for: The Clinical Characteristics and Prediction Nomograms for Primary Spine Malignancies
Source: Front Oncol. 2021 Feb 26;11:608323. doi: 10.3389/fonc.2021.608323 (PMC7959809; doi:10.3389/fonc.2021.608323)

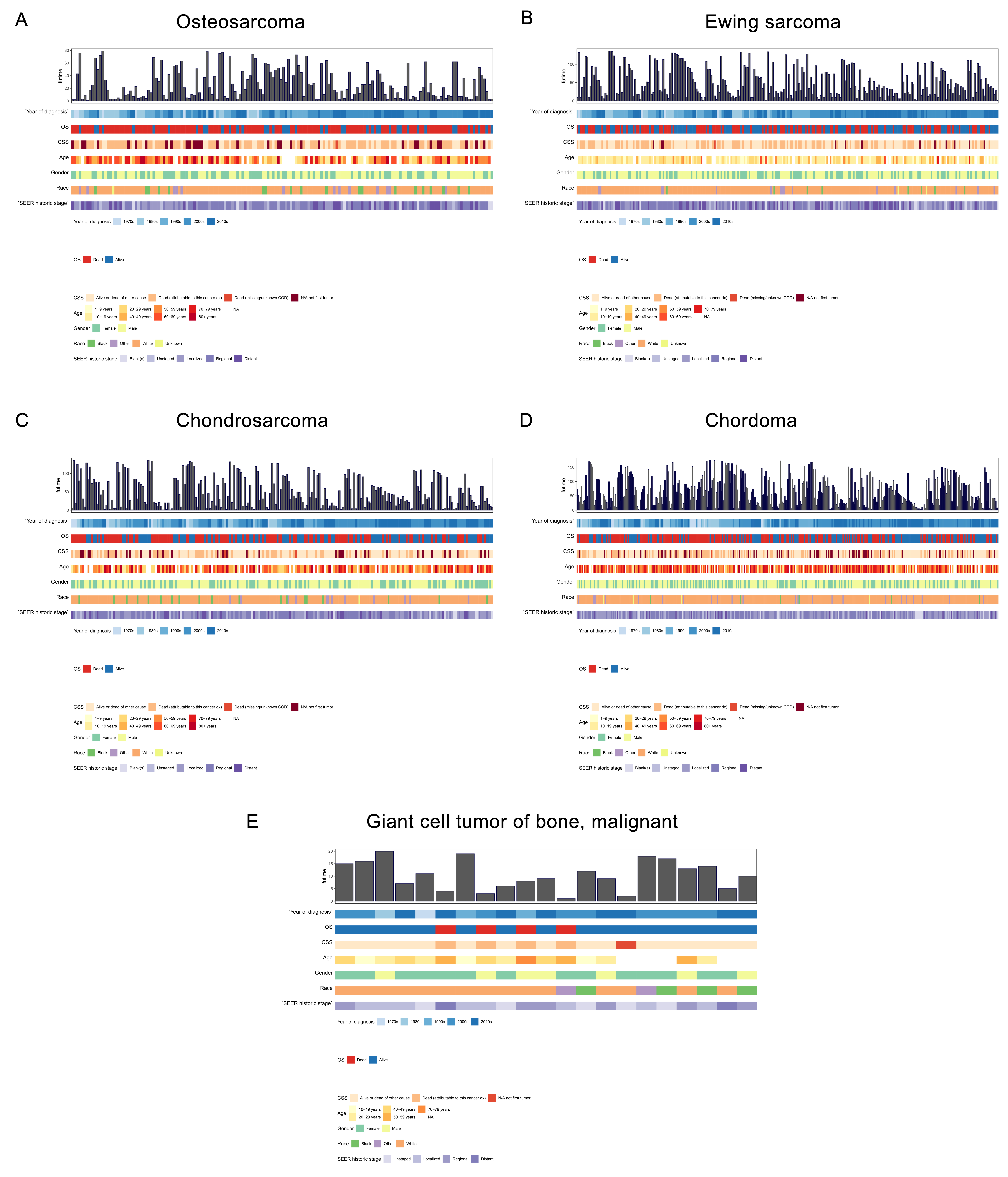

Supplement: Supplementary Figure 1 — The epidemiological analysis of patients with different types of PSMs. The integrated bar-plot and heatmap of demographics, tumor information, and patient outcomes of patients with osteosarcoma (A), Ewing sarcoma (B), chondrosarcoma (C), chordoma (D) and malignant GCTB (E). PSMs, primary spine malignancies; GCTB, Giant cell tumor of bone. [file Image_1.jpeg]

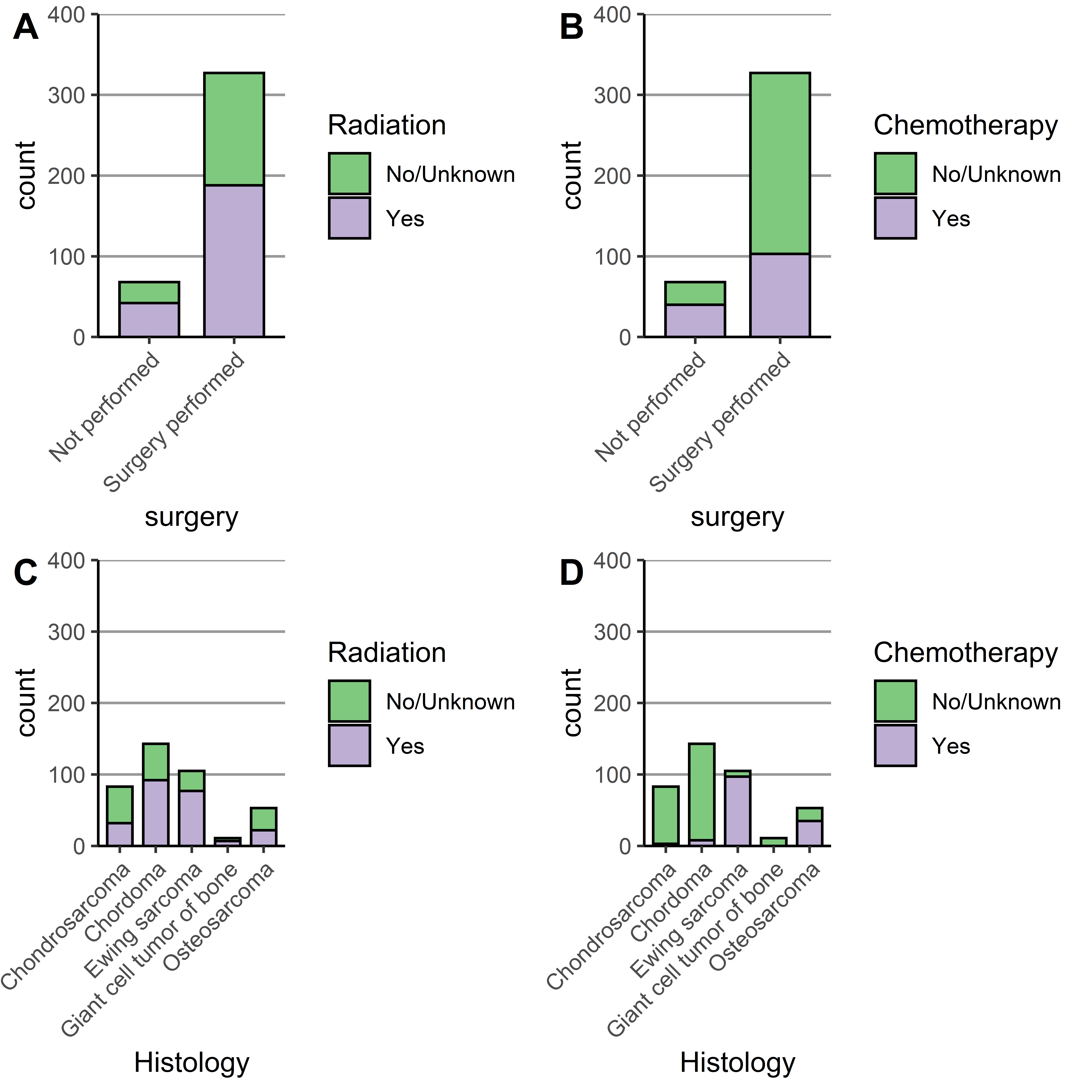

Supplement: Supplementary Figure 2 — The subgroup analysis between radiotherapy/chemotherapy and surgery/tumor histology. The bar graph revealed the correlation between radiotherapy and surgery (A), between radiotherapy and tumor histology (B), between chemotherapy and surgery (C), between chemotherapy and tumor histology (D). [file Image_2.tiff]
