# Supplementary material for: The Clinical Characteristics and Prediction Nomograms for Primary Spine Malignancies
Source: Front Oncol. 2021 Feb 26;11:608323. doi: 10.3389/fonc.2021.608323 (PMC7959809; doi:10.3389/fonc.2021.608323)
Supplement: Supplementary file 7 [file DataSheet_1.docx]

**Table S1** The subgroup analysis results between radiotherapy and surgery

| Radiotherapy | Surgery information | OR | 95%CI | P value |
| --- | --- | --- | --- | --- |
| Yes | Surgery not performed | 1.00 (reference) |  |  |
|  | Surgery performed | 0.84 | 0.49-1.42 | 0.516 |

**NOTE. No/Unknown radiation recode is the reference group**

**Abbreviation:** OR, odds ratio; CI, confidence interval.

*P < 0.05

**Table S2** The subgroup analysis results between chemotherapy and surgery

| Chemotherapy | Surgery information | OR | 95%CI | P value |
| --- | --- | --- | --- | --- |
| Yes | Surgery not performed | 1.00 (reference) |  |  |
|  | Surgery performed | 0.32 | 0.19-0.55 | <0.001* |

**NOTE. No/Unknown chemotherapy recode is the reference group**

**Abbreviation:** OR, odds ratio; CI, confidence interval.

*P < 0.05

**Table S3** The subgroup analysis results between radiotherapy and ICD-O-3 histology

| Radiotherapy | ICD-O-3 histology | OR | 95%CI | P value |
| --- | --- | --- | --- | --- |
| Yes | Chondrosarcoma | 1.00 (reference) |  |  |
|  | Chordoma | 2.88 | 1.64-5.03 | <0.001* |
|  | Ewing sarcoma | 4.38 | 2.36-8.13 | <0.001* |
|  | Giant cell tumor of bone | 2.79 | 0.76-10.29 | 0.124 |
|  | Osteosarcoma | 1.13 | 0.56-2.28 | 0.731 |

**NOTE. No/Unknown radiation recode is the reference group**

**Abbreviation:** OR, odds ratio; CI, confidence interval.

*P < 0.05

**Table S4** The subgroup analysis results between chemotherapy and ICD-O-3 histology

| Chemotherapy | ICD-O-3 histology | OR | 95%CI | P value |
| --- | --- | --- | --- | --- |
| Yes | Chondrosarcoma | 1.00 (reference) |  |  |
|  | Chordoma | 1.58 | 0.41-6.13 | 0.508 |
|  | Ewing sarcoma | 323.33 | 83.03-1259.16 | <0.001* |
|  | Giant cell tumor of bone | 6.27E-07 | 0.00-Inf | 0.990 |
|  | Osteosarcoma | 51.85 | 14.34-187.46 | <0.001* |

**NOTE. No/Unknown chemotherapy recode is the reference group**

**Abbreviation:** OR, odds ratio; CI, confidence interval.

*P < 0.05
